# The co-occurrence of multidimensional loneliness with depression, social anxiety and paranoia in non-clinical young adults: A latent profile analysis

**DOI:** 10.3389/fpsyt.2022.931558

**Published:** 2022-09-14

**Authors:** Anson Kai Chun Chau, Suzanne Ho-wai So, Xiaoqi Sun, Chen Zhu, Chui-De Chiu, Raymond C. K. Chan, Patrick W. L. Leung

**Affiliations:** ^1^Department of Psychology, The Chinese University of Hong Kong, Hong Kong, Hong Kong SAR, China; ^2^Department of Psychology, School of Educational Sciences, Hunan Normal University, Changsha, China; ^3^Cognition and Human Behavior Key Laboratory of Hunan Province, Hunan Normal University, Changsha, China; ^4^Neuropsychology and Applied Cognitive Neuroscience Laboratory, CAS Key Laboratory of Mental Health, Institute of Psychology, Chinese Academy of Sciences, Beijing, China; ^5^Department of Psychology, The University of Chinese Academy of Sciences, Beijing, China

**Keywords:** perceived social isolation, loneliness, depression, social anxiety, paranoia, core schemas

## Abstract

**Introduction:**

Loneliness is a negative experience arising from a mismatch between perceived and actual social relationships. Several dimensions of loneliness have been suggested, namely intimate, relational and collective loneliness. Loneliness has been linked to poorer mental health, with its co-occurrence with depression, social anxiety, and paranoia most widely reported. While expressions of these symptoms are heterogeneous across individuals in the non-clinical population, it remains unclear how these symptoms co-occur with one another and with various dimensions of loneliness. It is also of interest how trait factors such as core schemas about self/others may moderate these relationships between loneliness and co-occurring symptoms.

**Methods:**

A demographically diverse sample of young adults was recruited from multiple sources. The validated sample consisted of 2,089 participants (68.4% female), who completed an online survey consisting of questionnaires assessing levels of multidimensional loneliness, depression, social anxiety, paranoia, core schemas, and demographic characteristics. Latent profile analysis (LPA) was used to identify distinct profiles of loneliness and the three symptoms. Positive and negative core schemas about self and others were modeled as predictors of these profiles.

**Results:**

Five distinct profiles were identified. Profile 1 had low levels across all symptoms and dimensions of loneliness (*n* = 1,273, 60.9%). Profiles 2–5 were elevated on dimensions of loneliness, and were heightened in depression (*n* = 189, 9.0%), social anxiety (*n* = 206, 9.9%), paranoia (*n* = 198, 9.5%), and all symptoms (*n* = 223, 10.7%), respectively. Relative to Profile 1, the other four profiles scored higher on negative-self (adjusted *ORs* = 1.36–1.49, *ps* < 0.001) and negative-other schemas (adjusted *ORs* = 1.24–1.44, *ps* < 0.001), and lower on positive-self (adjusted *ORs* = 0.82–0.85, *ps* < 0.001) and positive-other schemas (adjusted *ORs* = 0.81–0.90, *ps* < 0.001).

**Conclusion:**

More marked intimate, relational and collective loneliness were evident across profiles that had heightened depression, social anxiety and/or paranoia, suggesting that loneliness may serve as a general risk factor for these psychopathologies. Our findings shed light on the heterogeneity of the co-occurrence of loneliness and various mental health difficulties in non-clinical young adults. Core schemas are suggested to be putative psychological mechanisms underlying their co-occurrence and even development.

## Introduction

Loneliness is a negative experience arising from the mismatch between perceived and actual social relationships (1). As a subjective experience, loneliness is conceptually distinct from objective social isolation, with its indicators such as the size of social network and frequency of social contact only weakly or moderately correlated with loneliness [e.g., (2)]. Loneliness is found to be correlated with various sociodemographic adversities (e.g., low socioeconomic status), as well as poor physical and mental health [e.g., (3, 4)]. Emerging evidence has suggested loneliness as a multidimensional phenomenon, with intimate, relational and collective dimensions consistently identified in samples of various age periods and cultures (5) and across measures of loneliness (6). Intimate (or emotional) loneliness indicates a feeling of aloneness and an absence of emotional support from close and significant others (6, 7). Relational (or social) loneliness refers to the lack of perceived closeness with and support from friends and relatives; whereas collective loneliness concerns identification and cohesion with social groups and society (e.g., civic groups and neighborhood organizations) (6, 7). These three dimensions are differentially associated with aspects of social relationships and indicators of wellbeing [e.g., (5, 8, 9)], supporting the utility and validity of multi-dimensionality of loneliness.

Loneliness is experienced as mild and transient for most people. However, for some individuals, loneliness could prolong and lead to negative physical and mental health consequences (7, 10, 11). Compared to the general population, more marked loneliness was reported by individuals with mental disorders, in particular major depressive disorder and social anxiety disorder (12). Loneliness in psychotic disorders has recently been examined. There is increasing evidence supporting a robust association with paranoia (13–15), one of the cardinal psychotic symptoms characterized by fears that others are targeting one for harm (16). Symptoms of major depressive disorder, social anxiety disorder, and psychotic disorders can be expressed below their clinical threshold in the non-clinical population and predispose the transition into a full-blown disorder (16–18).

Previous studies have reported that loneliness co-occurs with symptoms of depression, social anxiety and paranoia in non-clinical or general population samples [e.g., (14, 19, 20)]. Longitudinal studies also found that loneliness predicts an increase in the severity of these symptoms over time [e.g., (14, 21, 22)], suggesting the role of loneliness in their development and maintenance. However, this line of research has encountered two theoretical and methodological challenges. Firstly, most of these studies considered loneliness as a unidimensional construct. A few exceptions examined the relationships of depression and social anxiety with only emotional and social loneliness [e.g., (23–26)], lending preliminary support for a stronger association with emotional loneliness. So far, it is uncertain how the three dimensions of loneliness are distinctly related to these three symptoms. This line of research would benefit from examining intimate, relational and collective loneliness together in a single study.

Secondly, even among non-patients, it is common for symptoms of depression, social anxiety, and paranoia to be present together [e.g., (14, 27, 28)]. Various patterns of co-occurrence of these psychopathologies could exist, which are yet to be examined. In view of the potentially heterogeneous expressions of both loneliness dimensions and these symptoms in non-clinical individuals, a more comprehensive investigation of the relationship between loneliness and psychopathologies can be performed by using latent profile analysis (LPA), a person-centered statistical modeling approach that reveals distinct profiles of individuals based on pre-defined variables (29). By revealing profiles characterized by varying levels of multidimensional loneliness and psychopathologies, LPA helps to address the following question: whether loneliness dimensions are exacerbated in individuals with elevated symptom(s) exclusively, or is loneliness reported by individuals regardless of their levels of these symptoms. Addressing this research question is crucial to the understanding of distinctive patterns of co-occurrence and even development of loneliness and these symptoms in non-clinical populations, which has not been considered in the literature.

An LPA that takes into account the co-occurrence of loneliness and non-clinical symptoms will pave the way for addressing putative psychological mechanisms that may contribute to these phenomena. Core schemas are global and stable beliefs about the self and others (30, 31). Negative-self schemas (e.g., “I am bad and inferior”) are suggested to drive and maintain depression (32) and social anxiety (33). Negative-self (e.g., “I am vulnerable and weak”) and – other (e.g., “Others are hostile and untrustworthy”) schemas are proposed to predispose paranoid thinking (34, 35). Moreover, negative views about self and others are also proposed to maintain loneliness over time (1). On the contrary, the role of positive schemas is less understood. Freeman et al. (36) found that positive-self schemas were more strongly associated with social anxiety than paranoia.

The aim of the present study was twofold: (1) to identify profiles of co-occurrence of loneliness dimensions with depression, social anxiety, and paranoia; and (2) to examine the contribution of positive and negative core schemas of self and others in predicting these profiles. For (1), we sought to identify profiles of individuals based on validated measures of multidimensional loneliness, depression, social anxiety and paranoia. For (2), we would expect negative-self schemas to be more marked in profile(s) with elevated loneliness, depression, and/or social anxiety, whereas both negative-self and negative-other schemas would be more marked in profile(s) with elevated paranoia. Levels of positive-self and positive-other schemas were also compared across profiles. The current study focused on early adulthood, as previous studies have suggested that this life stage is among the most vulnerable to the emergence of loneliness (37–41) as well as the symptoms of interest (e.g., (42–44)).

## Materials and methods

Ethics approval for this study was granted by the Survey and Behavioral Research Ethics Committee of The Chinese University of Hong Kong. Written consent was obtained from all participants.

### Participants

Participants were Hong Kong residents aged 18–30. As this study focused on loneliness among non-patients, participants who reported a current or previous psychiatric diagnosis and those who were on psychiatric medication were excluded. Participants who reported any of the following neurological conditions, such as epilepsy and Tourette disorder etc., were also excluded. To reach participants with diverse socioeconomic backgrounds, recruitment was carried out through various means, including invitation through a marketing company, university mass mailing, distribution of leaflets at multiple locations around Hong Kong (e.g., public transport), promotion on social media platforms (i.e., Facebook and Instagram), and snowball sampling. The sampling procedure was detailed in Chau et al. (45).

### Measures

#### Indicator variables for latent profile analysis

Multidimensional loneliness was measured by the University of California, Los Angeles, Loneliness Scale (version 3) [UCLA-LS-v3, (46)]. It consists of 20 items assessing the frequency of experience of loneliness. Each item is rated on a 4-point scale ranging from 1 (“Never”) to 4 (“Often”). Dimension scores of intimate, relational and collective loneliness were computed according to (6), which have been validated in a Chinese sample of young adults (5). In the current sample, the internal consistencies of the loneliness dimension scores were 0.72, 0.83, and 0.75 respectively.

Depression was measured by the Patient Health Questionnaire-9 [PHQ-9, (47)]. The PHQ-9 consists of nine items based on the DSM-IV diagnostic criteria for depressive disorders. Each item is rated on a 4-point scale, ranging from 0 (“not at all”) to 3 (“nearly everyday”). The Chinese version of the PHQ-9 yielded satisfactory internal reliability (α = 0.82) and good construct validity (48). The internal consistency of the PHQ-9 total score in the current sample was 0.87.

Social anxiety was measured by the Social Interaction Anxiety Scale and Social Phobia Scale—Short Form [SIAS-6/SPS-6, (49)]. The SIAS-6/SPS-6 is a 12-item 5-point (0–4) rating scale assessing anxiety arising from social interactions and scrutiny by others. The Chinese version was translated for this study (unpublished). The SIAS-6/SPS-6 had excellent internal consistency in the current sample (Cronbach’s alpha = 0.92).

The Revised Green Paranoid Thoughts Scale [R-GPTS; (50)] is an 18-item 5-point (0–4) rating scale assessing ideas of reference (eight items) and ideas of persecution (10 items) in the general population. The Chinese version of the R-GPTS has been validated (51, 52). The R-GPTS had excellent internal consistency in the current sample (Cronbach’s alpha = 0.95).

#### Predictors of estimated profiles

The Brief Core Schema Scales [BCSS, (53)] is a 24-item 5-point (0–4) scale that assesses evaluative beliefs about the self and others. The BCSS yields four subscores (six items each): negative-self, positive-self, negative-other, and positive-other schemas. The Chinese version of the BCSS has been used in So et al. (51). The BCSS had good internal consistency in the current sample (Cronbach’s alphas of subscores > 0.79).

Participants also provided the following demographic information: age, gender, educational attainment, employment status, and monthly household income.

### Procedure

Consented participants completed an online survey individually, which consisted of the above self-report measures. Participants received a remuneration of HK$50 (US$6.41) upon completion of the online survey. Data collection was conducted from June to July 2018. Data validity was thoroughly checked according to recommendations in Curran (54). Specifically, response validity was evaluated with the attention check items (passing more than half of the attention check items) and long-string responses based on the UCLA-LS-v3. The procedure of data collection and data validity check was reported in Chau et al. (45).

### Data analysis

Descriptive statistics and correlations of multidimensional loneliness, depression, social anxiety, and paranoia were calculated in SPSS (55), whereas the LPA was performed on Mplus (56). The dimension scores of the UCLA-LS-v3 and total scores of the PHQ-9, SIAS-6/SPS-6, and R-GPTS were transformed into z-scores before being analyzed with the LPA. Latent profile models were estimated with robust maximum likelihood estimation with 500 initial stage random starts and 20 final stage optimizations, respectively. The LPA by default assumes that indicator variables are normally distributed and uncorrelated after conditioning on the latent profile membership (i.e., conditional independence), as well as homogeneity of variances across latent profiles (57). Given the robust associations between loneliness and these symptoms [e.g., (14, 15, 20)], we relaxed the assumption of conditional independence and allowed the indicator variables to be correlated. Given that our indicator variables are non-normal, we estimated the latent profile model using the *t*-distribution (58).

Models of two to eight profiles were estimated and compared on multiple criteria to determine the optimal number of profiles. These criteria included Akaike information criteria (AIC), Bayesian information criteria (BIC), Bozdogan’s consistent AIC (CAIC), sample size adjusted BIC (ssBIC), classification likelihood information criterion (CLC), normalized entropy criterion (NEC), entropy, and integrated complete likelihood BIC (ICL-BIC). A better-fitted model is indicated by a smaller value on all these criteria except for entropy, where a greater value would suggest a clear profile separation (59). The Lo-Mendell-Rubin likelihood ratio test was also performed to evaluate the relative model fit between K-profile and K-1 profile models. After identification of the optimal number of profiles, individuals were classified into the most likely profiles according to their posterior probabilities. To examine the differences in demographic characteristics and levels of indicator variables between profiles, chi-square tests and Kruskal-Wallis ANOVAs were used for categorical and continuous variables, respectively.

To examine research question 2, core schemas and demographic characteristics were compared across profiles using Vermunt’s (60) three-step approach. This approach fully accounts for any classification error and can simultaneously estimate the LPA and the effects of hypothesized predictors on profiles using a multinomial logistic regression approach. Self and other schemas (i.e., negative-self, positive-self, negative-other, positive-other) were treated as predictors of profiles, first without and then with demographic characteristics as covariates.

## Results

### Sample characteristics

A total of 2,595 participants responded to the survey, among whom 316 did not meet the recruitment criteria and were excluded (lack of contact: *n* = 134, repeated responses: *n* = 25, self-reported psychiatric or neurological conditions/use of psychiatric medication: *n* = 157). Another 190 participants were excluded for failing to meet the validity criteria (attention check items: *n* = 79; long-string responses: *n* = 111). The final sample consisted of 2,089 participants, among whom 527 (25.2%) were recruited through a marketing company. The mean age of the current sample was 23.63 years (*SD* = 3.67), with the majority being female (*n* = 1,429, 68.4%). Demographic characteristics of the sample were reported in full in Chau et al. (45). Means and correlations of key variables are reported in Table 1.

**TABLE 1:** Descriptive statistics of and correlations between indicator variables.

	*M*	*SD*	1	2	3	4	5	6
1. Depression	7.09	5.13	/					
2. Social anxiety	11.13	9.02	0.51	/				
3. Paranoia	14.94	13.30	0.58	0.62	/			
4. Intimate loneliness	10.16	2.42	0.47	0.55	0.47	/		
5. Relational loneliness	9.06	2.67	0.31	0.37	0.27	0.44	/	
6. Collective loneliness	8.61	2.34	0.31	0.42	0.29	0.44	0.69	/

All correlations are significant at p < 0.001.

### Latent profile analysis

For models with two to five profiles, the model optimization was satisfactory, and their best likelihood ratios were replicated. However, the best likelihood ratios for models with six to eight profiles were not replicated, so their parameters may not be reliably estimated.

As shown in Table 2, the six-profile model was indicated by BIC and CAIC, whereas the two-profile model was indicated by CLC, NEC, entropy, and ICL-BIC. The seven- and eight-profile model was indicated by ssBIC and AIC, respectively. The Lo-Mendell-Rubin likelihood ratio test suggested an optimal fit for models with five profiles or fewer. Examination of model parameters in the eight-, seven-, and six-profile models revealed that some parameters were not reliably estimated for interpretation. Upon consideration of model fit indexes, model parsimony, and proportion of individuals per profile (>5%) (61), a five-profile model was selected as the optimal solution.

**TABLE 2:** Model fit indexes for latent profile models.

No. of profiles	LL	AIC	BIC	CAIC	ssBIC	CLC	NEC	Entropy	ICL-BIC	LRT
2	−14858.22	29788.44	29991.64	30027.64	29877.26	30159.52	0.75	0.85	30434.72	<0.001
3	−14752.92	29593.83	29842.19	29886.19	29702.40	30217.28	0.89	0.85	30553.64	<0.001
4	−14657.18	29418.36	29711.87	29763.87	29546.66	30304.78	1.00	0.83	30702.29	0.036
5	−14580.50	29281.00	29619.67	29679.67	29429.04	30532.75	1.20	0.80	30991.41	<0.001
6	−14518.50	29173.00	29556.82	29624.82	29340.78	30376.99	1.06	0.82	30896.81	0.067
7	−14492.96	29137.91	29566.89	29642.89	29325.43	31343.62	1.79	0.71	31924.60	0.280
8	−14477.97	29123.94	29598.07	29682.07	29331.19	31423.30	1.83	0.72	32065.43	0.720

LL, log-likelihood value; AIC, Akaike information criteria; BIC, Bayesian information criteria; CAIC, Bozdogan’s consistent AIC; ssBIC, sample-size adjusted BIC; CLC, classification likelihood information criterion; NEC, normalized entropy criterion; ICL-BIC, integrated complete likelihood BIC; LRT, Lo-Mendell-Rubin likelihood ratio test.

The resultant five profiles consisted of 1,273 (60.9%), 189 (9.0%), 206 (9.9%), 198 (9.5%), and 223 (10.7%) participants, respectively. Demographic characteristics across profiles are reported in Table 3. There were significant differences in age, gender, educational attainment, employment status, and monthly household income across profiles (*ps* < 0.01). In particular, Profile 3 was younger and less likely to be full-time employed than Profile 1. Profile 5 was more male-dominant, followed by Profile 4 and then Profile 3, with Profiles 2 and 1 not differing from each other. As opposed to Profile 1, Profile 5 was less likely to receive a bachelor’s degree and earned the lowest monthly household income.

**TABLE 3:** Descriptive statistics of demographic characteristics, symptoms, and loneliness dimensions within identified profiles.

	Profiles					Differences across profiles^a^	*Post hoc* tests^b^
	1	2	3	4	5		
Number of individuals: *n* (%)	1,273 (60.9%)	189 (9.0%)	206 (9.9%)	198 (9.5%)	223 (10.7%)		
Demographic characteristics: *n* (%)							
Age	23.91 (3.64)	23.63 (3.48)	22.71 (3.57)	23.30 (3.80)	23.22 (2.79)	λ^2^(4) = 25.31, *p* < 0.001, ε^2^ = 0.01	1 > 3
Gender						λ^2^(4) = 34.05, *p* < 0.001, *V* = 0.13	
Female	909 (71.4%)	140 (74.1%)	137 (66.5%)	123 (62.1%)	120 (53.8%)		%: 2 = 1 > 3 > 4 > 5
Male	364 (28.6%)	49 (25.9%)	69 (33.5%)	75 (37.9%)	103 (46.2%)		%: 5 > 4 > 3 > 1 = 2
Educational attainment*						λ^2^(12) = 60.09, *p* < 0.001, *V* = 0.10	
Secondary education or below	315 (25.0%)	50 (26.6%)	73 (35.6%)	69 (35.0%)	86 (38.9%)		%: 5 > 1
Associate degree or higher diploma	226 (17.9%)	33 (17.6%)	45 (22.0%)	43(21.8%)	53 (24.0%)		/
Bachelor degree	614 (48.7%)	97 (51.6%)	84 (41.0%)	76 (38.6%)	73 (4.6)		%: 2 = 1 > 5
Master degree or above	105 (8.3%)	8 (4.3%)	3 (1.5%)	9 (4.6%)	9 (4.1%)		%: 1 > 3
Employment status**						λ^2^(16) = 33.26, *p* = 0.007, *V* = 0.06	
Full-time employment	731 (57.6%)	105 (55.9%)	80 (38.8%)	108 (55.1%)	117 (52.7%)		%: 1 = 2 = 4 > 3
Part-time employment (full-time student)	333 (26.3%)	49 (26.1%)	77 (37.4%)	53 (27.0%)	64 (28.8%)		/
Part-time employment (not full-time student)	47 (3.7%)	9 (4.8%)	14 (6.8%)	9 (4.6%)	12 (5.4%)		/
Not working and searching for job	76 (6.0%)	10 (5.3%)	23 (11.2%)	14 (7.1%)	14 (6.3%)		/
Not working and not searching for job	81 (6.4%)	15 (8.0%)	12 (5.8%)	12 (6.1%)	15 (6.5%)		/
Monthly household income						λ^2^(16) = 39.00, *p* = 0.001, *V* = 0.07	
<HKD 10,000	75 (5.9%)	19 (10.1%)	16 (7.8%)	23 (11.6%)	32 (14.3%)		%: 5 > 1
HKD 10,000–29,999	512 (40.2%)	75 (39.7%)	92 (44.7%)	73 (36.9%)	90 (40.4%)		/
HKD 30,000–49,999	405 (31.8%)	62 (32.8%)	65 (31.6%)	58 (29.3%)	74 (33.2%)		/
HKD 50,000–99,999	240 (18.9%)	27 (14.3%)	29 (14.1%)	36 (18.2%)	25 (11.2%)		%: 1 > 5
>HKD 100,000	41 (3.2%)	6 (3.2%)	4 (1.9%)	8 (4.0%)	2 (0.9%)		/
Indicator variables: *M* (*SD*)							
Symptoms							
Depression	4.42 (2.84)	14.27 (3.52)	7.83 (3.69)	8.79 (3.94)	14.04 (5.35)	χ^2^(4) = 982.22, *p* < 0.001, ε^2^ = 0.47	5 = 2 > 3 = 4 > 1
Social anxiety	6.41 (4.63)	10.52 (6.05)	24.18 (5.27)	12.06 (5.23)	25.77 (7.76)	χ^2^(4) = 1106.40, *p* < 0.001, ε^2^ = 0.53	5 = 3 > 2 = 4 > 1
Paranoia	7.96 (5.96)	12.48 (7.73)	16.24 (7.45)	32.04 (7.37)	40.48 (10.38)	χ^2^(4) = 1137.04, *p* < 0.001, ε^2^ = 0.55	5 = 4 > 3 > 2 > 1
Loneliness							
Intimate loneliness	9.33 (2.06)	10.68 (2.49)	11.89 (2.04)	11.62 (2.29)	11.52 (2.51)	χ^2^(4) = 411.09, *p* < 0.001, ε*^2^* = 0.20	5 = 4 = 3 > 2 > 1
Relational loneliness	8.28 (2.48)	10.06 (2.69)	10.76 (2.23)	10.11 (2.49)	10.12 (2.53)	χ^2^(4) = 280.59, *p* < 0.001, ε^2^ = 0.13	3 = 4 = 5 > 1 3 > 2 > 1
Collective loneliness	7.87 (2.09)	9.20 (2.34)	9.96 (2.02)	9.81 (2.08)	9.97 (2.46)	χ^2^(4) = 333.62, *p* < 0.001, ε^2^ = 0.16	5 = 3 > 2 > 1 4 > 1

*Valid N = 2,071, ** valid N = 2,080. ^a^Kruskal-Wallis one-way ANOVA for continuous variables, chi-square test for categorical variables. ^b^Games-Howell post hoc test with Bonferroni adjustment for continuous variables, pairwise Z-test with Bonferroni adjustment for categorical variables. Only significant pairwise comparisons are reported.

**FIGURE 1:**
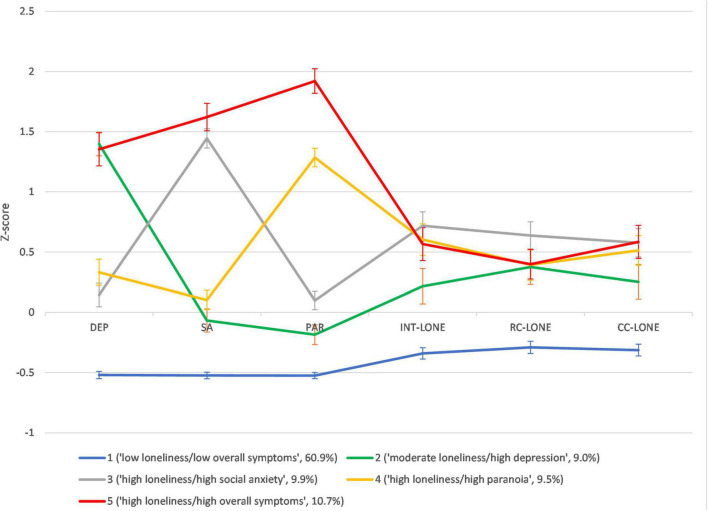
Z-score profiles and 95% confidence interval of psychopathologies and loneliness dimensions across five profiles. DEP, depression; SA, social anxiety; PAR, paranoia; INT-LONE, intimate loneliness; RC-LONE, relational loneliness; CC-LONE, collective loneliness.

Means and SDs of the indicator variables of the five profiles are shown in Table 3 and Figure 1. Kruskal-Wallis ANOVAs revealed significant differences in the levels of all indicator variables (*ps* < 0.001, ε*^2^* = 0.47–0.55) and dimensions of loneliness (*ps* < 0.001, ε*^2^* = 0.13–0.20) across profiles. Profile 1 scored the lowest on depression, social anxiety, paranoia and the three dimensions of loneliness; hence, it was labeled as “low loneliness/low overall symptoms.” Profiles 2–5 reported higher levels of dimensions of loneliness than Profile 1. In particular, levels of intimate loneliness were comparable between Profiles 3–5, which were higher than Profile 2. The level of relational loneliness was the highest in Profile 3, which was comparable to Profiles 4 and 5 but higher than Profile 2. The level of collective loneliness was the highest in Profiles 5 and 3, which were higher than Profile 2. In addition, while Profile 5 was elevated on all symptoms, Profiles 2–4 were characterized by elevated levels of depression, social anxiety, or paranoia, respectively. Therefore, Profiles 2–4 were labeled as “moderate loneliness/high depression,” “high loneliness/high social anxiety,” and “high loneliness/high paranoia,” respectively, and Profile 5 was labeled as “high loneliness/high overall symptoms.”

### Prediction of profiles with core schemas as predictors

**FIGURE 2:**
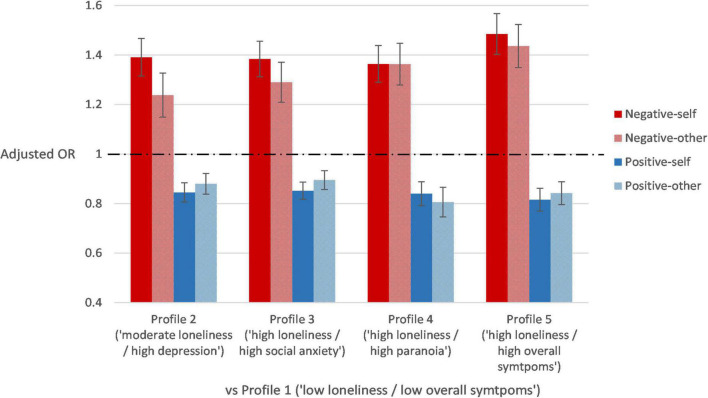
Adjusted odds ratios and the 95% confidence intervals of the prediction of profiles by core schemas after adjusting for demographic characteristics.

Results of multinomial logistic regression of latent profiles on all BCSS scores are shown in Table 4. Figure 2 displays the results of the regression models with adjustment for demographic characteristics. A higher BCSS negative-self schema score increased the likelihood of being classified in Profiles 2–5 as opposed to Profile 1 (*ORs*: 1.34–1.47, *ps* < 0.001), even after adjusting for demographic characteristics (adjusted *ORs*: 1.36–1.49, *ps* < 0.001). The odds ratios for Profile 5 as opposed to other profiles were significant (adjusted *ORs*: 1.07–1.49, *ps* < 0.010). Similarly, a higher BCSS negative-other schema score increased the likelihood of being classified in Profiles 2–5 as opposed to Profile 1 (*OR*s: 1.23–1.42, *ps* < 0.001; adjusted *ORs*: 1.24–1.44, ps < 0.001). The odds ratios for Profiles 4 and 5 as opposed to other profiles were significant (adjusted *ORs*: 1.05–1.44, *ps* < 0.050).

**TABLE 4:** Multinomial logistic regression of latent profiles using Vermunt’s 3-step approach.

		Bivariate associations			Adjusted for demographic characteristics^a^		
			
	Reference profile	*OR*	95% CI	*p*	Adjusted *OR*	95% CI	*p*
**Negative-self schemas**							
Profile 2	Profile 1	**1.38**	1.32–1.46	<0.001	**1.39**	1.32–1.47	<0.001
Profile 3	Profile 1	**1.37**	1.30–1.43	<0.001	**1.38**	1.32–1.46	<0.001
Profile 4	Profile 1	**1.34**	1.28–1.41	<0.001	**1.36**	1.29–1.44	<0.001
Profile 5	Profile 1	**1.47**	1.40–1.55	<0.001	**1.49**	1.41–1.57	<0.001
Profile 3	Profile 2	0.99	0.95–1.03	0.554	1.00	0.95–1.04	0.830
Profile 4	Profile 2	0.97	0.92–1.02	0.221	0.98	0.93–1.03	0.434
Profile 5	Profile 2	**1.06**	1.02–1.11	0.009	**1.07**	1.02–1.12	0.006
Profile 4	Profile 3	0.98	0.94–1.03	0.464	0.99	0.94–1.03	0.536
Profile 5	Profile 3	**1.08**	1.03–1.12	0.002	**1.07**	1.03–1.12	0.003
Profile 5	Profile 4	**1.09**	1.04–1.15	0.002	**1.09**	1.03–1.15	0.003
**Negative-other schemas**							
Profile 2	Profile 1	**1.23**	1.15–1.31	<0.001	**1.24**	1.15–1.33	<0.001
Profile 3	Profile 1	**1.28**	1.20–1.35	<0.001	**1.29**	1.21–1.37	<0.001
Profile 4	Profile 1	**1.35**	1.28–1.42	<0.001	**1.36**	1.28–1.45	<0.001
Profile 5	Profile 1	**1.42**	1.34–1.49	<0.001	**1.44**	1.36–1.52	<0.001
Profile 3	Profile 2	1.04	0.98–1.10	0.226	1.04	0.98–1.11	0.203
Profile 4	Profile 2	**1.10**	1.04–1.16	0.003	**1.10**	1.04–1.17	0.003
Profile 5	Profile 2	**1.16**	1.09–1.22	<0.001	**1.16**	1.10–1.23	<0.001
Profile 4	Profile 3	**1.06**	1.01–1.11	0.020	**1.06**	1.01–1.11	0.025
Profile 5	Profile 3	**1.11**	1.07–1.16	<0.001	**1.11**	1.06–1.17	<0.001
Profile 5	Profile 4	**1.05**	1.01–1.10	0.016	**1.05**	1.01–1.10	0.018
**Positive-self schemas**							
Profile 2	Profile 1	**0.85**	0.81–0.88	<0.001	**0.85**	0.81–0.88	<0.001
Profile 3	Profile 1	**0.86**	0.82–0.89	<0.001	**0.85**	0.82–0.89	<0.001
Profile 4	Profile 1	**0.84**	0.80–0.89	<0.001	**0.84**	0.80–0.89	<0.001
Profile 5	Profile 1	**0.81**	0.76–0.86	<0.001	**0.82**	0.77–0.86	<0.001
Profile 3	Profile 2	1.01	0.96–1.06	0.718	1.01	0.95–1.07	0.791
Profile 4	Profile 2	1.00	0.93–1.06	0.875	0.99	0.93–1.06	0.860
Profile 5	Profile 2	0.95	0.89–1.02	0.158	0.97	0.90–1.03	0.289
Profile 4	Profile 3	0.99	0.93–1.05	0.636	0.99	0.93–1.05	0.674
Profile 5	Profile 3	0.95	0.88–1.01	0.091	0.96	0.90–1.02	0.200
Profile 5	Profile 4	0.96	0.88–1.05	0.350	0.97	0.89–1.06	0.491
**Positive-other schemas**							
Profile 2	Profile 1	**0.88**	0.84–0.93	<0.001	**0.88**	0.84–0.92	<0.001
Profile 3	Profile 1	**0.90**	0.86–0.94	<0.001	**0.90**	0.86–0.93	<0.001
Profile 4	Profile 1	**0.82**	0.77–0.87	<0.001	**0.81**	0.75–0.87	<0.001
Profile 5	Profile 1	**0.84**	0.79–0.88	<0.001	**0.84**	0.80–0.89	<0.001
Profile 3	Profile 2	1.02	0.96–1.08	0.513	1.02	0.96–1.08	0.585
Profile 4	Profile 2	**0.93**	0.86–1.00	0.042	**0.92**	0.84–1.00	0.030
Profile 5	Profile 2	0.95	0.88–1.01	0.101	0.96	0.89–1.03	0.199
Profile 4	Profile 3	**0.91**	0.85–0.98	0.006	**0.90**	0.83–0.97	0.006
Profile 5	Profile 3	**0.93**	0.87–0.99	0.026	0.94	0.88–1.01	0.073
Profile 5	Profile 4	1.02	0.93–1.12	0.659	1.05	0.95–1.15	0.384

^a^Demographic characteristics include age, gender (1 = male, 0 = female), educational attainment, employment status (1 = full-time employed, 0 = not full-time employed), and monthly household income. BCSS, Brief Core Schema Scale. Statistically significant ORs are in bold typeface.

Higher BCSS positive-self and positive-other scores decreased the likelihood of being classified in Profiles 2–5 as opposed to Profile 1 (positive-self: *ORs*: 0.81–0.86, *ps* < 0.001; positive-other: *ORs*: 0.82–0.90, *ps* < 0.001). These effects remained robust after adjustment for demographic characteristics (positive-self: adjusted *ORs*: 0.82–0.85, *ps* < 0.001; positive-other: adjusted *ORs*: 0.81–0.90, *ps* < 0.001). Also, the effects of positive-other schemas for Profile 4 were significant as opposed to Profiles 2 (adjusted *OR*: 0.92, *p* = 0.030) and 3 (adjusted *OR*: 0.90, *p* = 0.006), but not to Profile 5 (adjusted *OR*: *0.96*, *p* = 0.364).

## Discussion

The current study examined the distinct patterns of co-occurrence of loneliness, depression, social anxiety, and paranoia in a non-clinical sample of young adults. This study built on and extended the current evidence of positive correlations between loneliness, depression, social anxiety, and paranoia (e.g., 14, 15, 20), and focused on young adulthood when these experiences are likely to have emerged in vulnerable individuals. Considering loneliness as a multidimensional phenomenon, this study identified distinct profiles of co-occurrence of loneliness and the three symptoms using LPA and compared these profiles on core schemas about self and others.

Our LPA revealed five profiles of individuals. The majority of the individuals (60.9%) were classified as Profile 1, scoring low on dimensions of loneliness and all symptoms. The remaining four profiles had moderate to high levels of loneliness and were elevated in at least one symptom. Although the current sample did not have a psychiatric diagnosis, a substantial proportion of them had elevated symptoms and loneliness. Profile 5, which reported high levels of loneliness and multiple symptoms, is likely to bring the most distress and functioning impairment among profiles (62). This finding speaks for the importance of early identification and intervention for distressing symptoms among non-patients [e.g., (63, 64)].

The LPA revealed two interesting patterns of the co-occurrence between loneliness and psychiatric symptoms. First, when loneliness is reported to be at least a moderate level (Profiles 2–5), it is always accompanied by elevated depression, social anxiety, paranoia, or their combination. There did not exist a profile that had elevated loneliness but low symptoms. Together with evidence that loneliness is associated with emotional instability and hypervigilance to social threats, which predispose the development of affective and psychotic disorders [e.g., (8, 21, 65)], and that there are polygenetic overlaps between loneliness with these disorders (66, 67), the current findings lend support to the idea that loneliness may be a general risk factor that pertains to various psychopathologies (68, 69).

Second, the results revealed fine distinctions in the levels of dimensions of loneliness across profiles, confirming the need to consider loneliness as a multidimensional construct. In particular, Profiles 3, 4, and 5 reported the highest levels of social anxiety and/or paranoia, as well as intimate loneliness, supporting their proximal relationships. This result is consistent with the recent findings that excessive worry in and a tendency to withdraw from intimate relationships (i.e., attachment insecurity 70) are characteristic of social anxiety (71), paranoia (72, 73), and intimate loneliness (74, 75). For relational and collective loneliness, their differences across profiles were more subtle, rendering interpretations more ubiquitous. Nevertheless, the current findings highlight the need to consider the fine-grained dimensions of loneliness (76, 77), paving the way for future research on the dimensionality of loneliness and its implications on the expression of psychopathologies.

As hypothesized, the levels of negative-self schemas were higher in profiles with elevated symptoms and loneliness (i.e., Profiles 2–5). The levels of negative-other schemas were also higher in profiles with elevated symptoms and loneliness, among which the levels in profiles with elevated paranoia only (i.e., Profiles 4 and 5) were more prominent. As exploratory analyses, positive-self and positive – other schemas were found to have effects in the opposite direction to negative-self and negative-other schemas. These findings suggested beliefs about the self as a shared psychological mechanism across profiles with elevated symptoms and loneliness, whereas beliefs about others as a specific psychological mechanism for paranoia [e.g., (34, 35, 53)]. Overall, core schemas may contribute to the heterogeneous patterns of expression of loneliness and various symptoms, with specific schemas impacting on the development of loneliness and various mental disorders in distinct ways. Interventions targeting maladaptive schemas have shown promise in improving depressive symptoms (78) and persecutory delusions (79) in clinical populations. These interventions may also benefit the non-clinical populations with elevated levels of symptoms and/or loneliness.

This study had several limitations. The main limitation is that the screening for psychiatric history was based on participants’ self-report, which was not verified by diagnostic interviews or medical records. Since it was an online survey study, no extra steps were taken to confirm participants’ reports. Besides, all measures of psychopathologies were self-reported. Although these questionnaires have satisfactory psychometric properties, they may not correspond perfectly with findings from interviewer-rated measures. In addition, the current study only considered core schemas as predictors of profiles. There may be other etiological processes, such as biases in reasoning and social cognition [e.g., (80–82)], that contribute to the classification of profiles. Lastly, the cross-sectional design of the study did not allow us to infer any directional relationships between loneliness and co-occurring symptoms, or to test the causal role of core schemas in the development of these profiles.

Against these caveats, the current study found that elevated loneliness, including the intimate, relational and collective dimensions, tends to co-occur with heightened depression, social anxiety, and/or paranoia in non-clinical young adults, suggesting loneliness as a general risk factor to these symptoms. Our results shed light on the distinct patterns of loneliness and various mental health difficulties and the contributions of positive and negative schemas underlying these patterns of co-occurrence.

## Data availability statement

The raw data supporting the conclusions of this article will be made available by the authors, without undue reservation.

## Ethics statement

The studies involving human participants were reviewed and approved by the Survey and Behavioral Research Ethics Committee (SBREC), The Chinese University of Hong Kong. The patients/participants provided their written informed consent to participate in this study.

## Author contributions

AC and SH-wS designed the study, wrote the protocol, and wrote the first draft of the manuscript. AC, SH-wS, XS, and CZ managed the data collection. C-DC, RC, and PL oversaw the data collection. AC undertook the statistical analysis. All authors contributed to and have approved the final manuscript.
